# Associations of Close Social Connections With Smoking and Vaping: A Population Study in England

**DOI:** 10.1093/ntr/ntae225

**Published:** 2024-09-24

**Authors:** Sarah E Jackson, Hazel Squires, Lion Shahab, Harry Tattan-Birch, Charlotte Buckley, Robin C Purshouse, Jamie Brown

**Affiliations:** Department of Behavioural Science and Health, University College London, London, UK; SPECTRUM Consortium, Edinburgh, UK; Sheffield Centre for Health and Related Research, University of Sheffield, Sheffield, UK; Department of Behavioural Science and Health, University College London, London, UK; SPECTRUM Consortium, Edinburgh, UK; Department of Behavioural Science and Health, University College London, London, UK; SPECTRUM Consortium, Edinburgh, UK; Department of Automatic Control and Systems Engineering, University of Sheffield, Sheffield, UK; Department of Automatic Control and Systems Engineering, University of Sheffield, Sheffield, UK; Department of Behavioural Science and Health, University College London, London, UK; SPECTRUM Consortium, Edinburgh, UK

## Abstract

**Introduction:**

Studies consistently demonstrate smoking is a socially contagious behavior, but less is known about the influence of social connections on vaping. This study examined associations between having close social connections who smoke or vape and relevant smoking and vaping outcomes.

**Aims and Methods:**

This was a representative cross-sectional survey of adults (≥16 years) in England. Participants (*n* = 1618) were asked how many people they discuss important matters with (ie, close social connections) and how many of them smoke/vape. We tested associations between (1) smoking and (2) vaping among close social connections and participants’ own smoking and vaping status; harm perceptions of e-cigarettes (among current smokers); attempts and success in quitting smoking (among past-year smokers); and use of e-cigarettes as a smoking cessation aid (among past-year smokers who tried to quit).

**Results:**

Adults with ≥1 close social connection who smoke were more likely than those with none to smoke themselves (32.8% vs. 9.4%; OR_adj_ = 7.23[95% CI: 4.74 to 11.0]) and had an uncertain lower likelihood to quit (12.2% vs. 19.8%; OR_adj_ = 0.46[0.17–1.23]). Those with ≥1 close social connection who vape were more likely than those with none to vape themselves (29.6% vs. 6.3%; OR_adj_ = 5.16[3.15–8.43]) and to use e-cigarettes in their most recent attempt to quit (57.0% vs. 27.9%; OR_adj_ = 18.0[1.80–181]), and had an uncertain higher likelihood to perceive e-cigarettes as less harmful than cigarettes (30.8% vs. 12.2%; OR_adj_ = 2.37[0.82–6.90]).

**Conclusions:**

In England, we replicated well-established associations with smoking and found similar evidence for vaping. People were much more likely to vape and to use e-cigarettes to quit smoking if they had close social connections who vaped.

**Implications:**

The cross-sectional design means it is not clear whether smoking/vaping among close social connections influences people to smoke/vape themselves, or whether people who smoke/vape select to form close social connections with others who similarly smoke/vape. Further research is required to establish causality. If the associations we observed are causal, interventions that encourage smokers to switch to vaping may have positive spillover effects on social connections’ perceptions of e-cigarettes and the use of these products to support smoking cessation.

## Introduction

Smoking is a socially contagious behavior. Substantial research has shown that people are much more likely to smoke if others in their social networks smoke (eg, their family, friends, or colleagues) and more likely to stop smoking if those in their social networks quit.^1–4^ Less is known about the influence of social connections on people’s perception and use of e-cigarettes (vaping). A large body of evidence shows vaping is both less harmful than smoking^5^ and effective in helping smokers quit.^6^ However, it is not risk-free.^5^ In England, there has been a recent and rapid rise in vaping prevalence since 2021, particularly among young adults,^7–9^ which has raised concerns about the initiation of vaping among people who would not otherwise have smoked. Up-to-date information on how social networks influence smoking and vaping, and the differences between the behaviors, can inform the extent to which interventions that encourage smokers to switch to vaping or discourage uptake of vaping among never-smokers will have knock-on effects on the people around them.

Social network influences might be similar for smoking and vaping because both behaviors are shaped by peer dynamics, social norms, and the desire for social conformity. Within social groups, observing peers engaging in smoking or vaping can lead to the adoption of these behaviors,^1,10,11^ especially among young people who are more susceptible to peer influence.^12^ Both behaviors are also influenced by perceived social rewards, such as fitting in or gaining social status.^13,14^ In addition, the spread of smoking and vaping behaviors is facilitated through shared environments and social settings where these activities are visible and can become normalized.^1,15^

Social network influences on smoking and vaping may also differ for several reasons. On the one hand, influences may be stronger for vaping as a newer behavior. According to the “diffusion of innovations” theory, diffusion is a process by which new ideas, innovations, or behaviors spread through a social network over time, involving adoption by different segments of the population and influence via communication channels and social systems.^16^ While smoking has been an established behavior for many decades, vaping first became established in England between 2011 and 2013^17^ and its prevalence has been rising rapidly in recent years,^8^ so it may have greater potential to spread across social connections. The lower risk profile of e-cigarettes than combustible tobacco^5^ may also make people more likely to take up vaping than smoking if their peers are doing the same—although widespread misperceptions of the relative harms may undermine such differences.^18^ Age differences in resistance to peer influence may also play a role, given the much higher prevalence of vaping than smoking in adolescence,^19^ when people are more easily influenced by those around them.^12^ Recent studies in the US have shown young people are more likely to take up vaping if their friends vape.^11,20^

On the other hand, social network influences could also be stronger for smoking, as a more established, conspicuous behavior with strong social meanings and values. Social and cultural norms play an important role in shaping smoking behavior.^21–23^ Smoking may persist more strongly than vaping within certain social groups because it is closely linked to emotive issues of class and family allegiance; not smoking may therefore be interpreted as a desire to distinguish oneself from the group and reject its values.^22^ Smoking is also a more visible behavior than vaping, which can be done more discreetly, which might exert different social pressures—although part of the culture around vaping, which relates to its social aspect, involves more conspicuous use of e-cigarettes (eg, “cloud chasing”).^24^ In addition, communication about cessation may play a more direct role in influencing smoking and quitting behaviors within social networks than it does for vaping. While e-cigarettes were initially viewed as a tool for smoking cessation, they are increasingly seen as a lifestyle product unrelated to tobacco smoking.^25^

Using data from a representative cross-sectional survey of adults in England, this study aimed to explore the potential influence of having close social connections to people who smoke or vape on relevant smoking and vaping outcomes. We aimed to address the following research questions (RQs):

1. What proportion of adults in England have close social connections who (1) smoke and (2) vape, and how does it differ by age, gender, and occupational social grade (as a marker of socioeconomic position)?
2. To what extent is having close social connections who smoke/vape associated with:
  a. Whether adults currently smoke/vape themselves;
  b. Current smokers’ harm perceptions of e-cigarettes compared with cigarettes;
  c. Whether past-year smokers (smokers or those who quit in the past year) attempt to quit smoking or succeed;
  d. Whether past-year smokers who attempt to quit smoking use e-cigarettes as a cessation aid?
3. Does RQ2a differ by age?
4. Are any associations in RQ1 and RQ2 stronger for those with more close social connections who smoke/vape (ie, is there a dose–response association)?

These research questions were developed a priori and preregistered. Given the large number of potential associations we could explore with the available data, we had to be selective. We, therefore, prioritized investigating associations between having close social connections who smoke/vape and engaging in the same behavior over exploring cross-behavior associations and exploring the extent to which associations differed by age (as a key variable associated with smoking, vaping, and social influence) over other potential sociodemographic moderators.

## Methods

### Preregistration

The study protocol and analysis plan were preregistered on Open Science Framework (https://osf.io/hvxcp/).

### Design

Data were drawn from the Smoking Toolkit Study, a monthly cross-sectional survey of a representative sample of adults in England.^26,27^ The study uses a hybrid of random probability and simple quota sampling to select a new sample of approximately 1700 adults aged ≥16 years each month. Data are collected via computer-assisted telephone interviews. Comparisons with other national surveys and sales data show key variables such as sociodemographic characteristics, smoking prevalence, and cigarette consumption are nationally representative.^26,28^

In November 2023, questions on current smoking and vaping among people with whom the participant discusses important matters were added to the survey for a single wave. The present study analyzed data from participants surveyed in this wave.

### Measures

#### Exposures

Smoking and vaping among close social connections were assessed among all participants with a series of questions. The first question asked: “How many people, if any, do you discuss matters that are important to you with nowadays?”—a standard phrasing used in elicitation of close associate ego-centric networks.^29,30^ Response options were none, 1, 2, 3, 4, 5, 6–9, 10–19, and ≥20. Follow-up questions asked those who reported discussing important matters with one person whether this person currently smokes or vapes and asked those who reported discussing important matters with more than one person how many of these people currently smoke or vape (with the same response options as the first question).

Our two exposures were (1) smoking among close social connections and (2) vaping among close social connections. For each of these, we analyzed three variables:

1. Having at least one close social connection who smokes/vapes, analyzed as a binary variable, yes/no.
2. The number of close social connections who smoke/vape, analyzed as an ordinal variable, with responses collapsed to 0, 1, 2, and ≥3 (because few participants reported having more than 3 connections who smoke/vape).
3. The proportion of close social connections who smoke/vape, calculated as the number of close social connections who smoke/vape divided by the total number of close social connections reported (for each variable, responses of 6–9 were imputed as 7.5, 10–19 as 14.5, and ≥20 as 20) and analyzed as an ordinal variable, with the following categories: 0%, >0%, and <50%, and ≥50% (ie, none, a minority, and the majority).

#### Outcomes

Smoking status was assessed among all participants by asking which of the following best applied to them:

a) I smoke cigarettes (including hand-rolled) every day.
b) I smoke cigarettes (including hand-rolled), but not every day.
c) I do not smoke cigarettes at all, but I do smoke tobacco of some kind (eg, pipe, cigar, or shisha).
d) I have stopped smoking completely in the last year.
e) I stopped smoking completely more than a year ago.
f) I have never been a smoker (ie, smoked for a year or more).

Those who responded *a-c* were considered current smokers (coded 1) and those who responded *e-f* were considered nonsmokers (coded 0). For analyses of quitting outcomes, those who responded *a-d* were considered past-year smokers and formed the analytic sample.

Vaping status was assessed among all participants with several questions asking about the use of a range of nicotine products. Current smokers were asked “Do you regularly use any of the following in situations when you are not allowed to smoke?” and those who reported cutting down “Which, if any, of the following are you currently using to help you cut down the amount you smoke?”; current smokers and those who had quit in the past year (ie, past-year smokers) were asked “Can I check, are you using any of the following either to help you stop smoking, to help you cut down or for any other reason at all?”; and nonsmokers were asked “Can I check, are you using any of the following?.” Those who reported using an e-cigarette in response to any of these questions were considered current vapers (coded 1), else they were considered non-vapers (coded 0).

Harm perceptions of e-cigarettes were assessed among current smokers only with the question: “Compared to regular cigarettes, do you think electronic cigarettes are more, less, or equally harmful to health?” Response options were “more harmful,” “less harmful,” “equally harmful,” or “don’t know.” We dummy-coded these response options for analysis as less harmful versus all other responses, consistent with current evidence that e-cigarettes are less harmful than cigarettes.^5^

Attempts to quit smoking were assessed among past-year smokers with the question: “How many serious attempts to stop smoking have you made in the last 12 months? By serious attempt I mean you decided that you would try to make sure you never smoked again. Please include any attempt that you are currently making and please include any successful attempt made within the last year”. Those who reported making at least one serious quit attempt in the past year were coded 1; otherwise, they were coded 0.

Smoking cessation was assessed among past-year smokers based on responses to the item assessing smoking status. Those who responded *d* (stopped smoking completely in the last year) were coded 1 and those who responded *a-c* (current smokers) were coded 0.

Use of e-cigarettes as a smoking cessation aid was assessed among past-year smokers who made ≥1 serious past-year quit attempt with the question: “Which, if any, of the following did you try to help you stop smoking during the most recent serious quit attempt?.” Participants were asked to indicate all that apply. Those who responded “electronic cigarette” were coded 1, else they were coded 0.

### Covariates

Analyses of all smoking and vaping outcomes were adjusted for the total number of close social connections the participant reported having (0, 1, 2, 3, 4, or ≥5), age, gender, and occupational social grade (ABC1 includes managerial, professional and upper supervisory occupations/C2DE includes manual routine, semi-routine, lower supervisory, long-term unemployed, and state pension).

Analyses of current vaping also adjusted for smoking status (current smoker [responses *a-c*], ex-smoker [responses *d*-*e*], never smoker [response *f*]). Analyses of harm perceptions of e-cigarettes among current smokers also adjusted for vaping status (current vaper/non-vaper). Analyses of smoking cessation and use of e-cigarettes as a smoking cessation aid among past-year smokers also adjusted for the level of cigarette addiction (indexed by self-reported strength of urges to smoke^31^).

### Statistical Analysis

Data were analyzed in R v.4.2.1. Missing cases were excluded on a per-analysis basis (see **Table S1** for numbers of missing cases on each variable). The Smoking Toolkit Study uses raking to weight the sample to match the population of England in terms of key demographics. These key demographics are determined each month using data from the UK Census, the Office for National Statistics mid-year estimates, and the National Readership Survey.^26^ All analyses used weighted data, analyzed using the *survey* package. The survey did not have a complex sampling design.

Among all adults and stratified by age, gender, and occupational social grade, we reported descriptive data on the total number of close social connections, the proportion with at least one close social connection who smokes/vapes, and the number and proportion of close social connections who smoke/vape.

We used logistic regression to test associations between the exposures and outcomes of interest. For each outcome, we constructed three models. Model 1 adjusted only for the total number of close social connections since participants with more connections will be more likely by chance to have at least one connection who is a smoker/vaper than participants with fewer connections. Model 2 additionally adjusted for covariates (as described in the “Covariates” section above), and Model 3 additionally adjusted for the other behavior among close social connections (ie, models testing associations with the number of close social connections who smoke were additionally adjusted for the number of close social connections who vape). This sequential modeling approach was intended to provide insight into the extent to which any differences in the outcomes between those who did/did not have close social connections who smoke/vape were explained by covariates and having close social connections who engaged in the other behavior. For associations with current smoking and current vaping, we repeated model 3 with the addition of the interaction with age, to explore whether associations between having at least one close social connection who smokes or vapes and participants’ own smoking and vaping behavior differed by age.

We followed the “New Statistics” approach to reporting and interpretation of results,^32,33^ focusing on effect sizes and confidence intervals rather than dichotomous thinking about the statistical significance (ie, whether a result is significant or not significant, based on an arbitrary threshold).

## Results

A total of 1659 adults aged ≥16 years were surveyed in November 2023. We excluded 41 from all analyses who responded that they did not know how many people, if any, they discussed important matters with, leaving a sample of 1618 participants for analysis. **Table S1** provides a summary of sample characteristics. The mean age was 48.0 years, 50.8% were women, and 43.7% were from less advantaged social grades.

### Descriptive Data on Smoking and Vaping Among Close Social Connections

**Table S2** provides data on the total number of close social connections participants reported. **Tables 1 and 2** provide data on smoking and vaping, respectively, among close social connections.

**Table 1.** Descriptive Data on Smoking Among Participants’ Close Social Connections

	At least one close social connection who smokes		Number of close social connections who smoke				Proportion of close social connections who smoke		
	No	Yes	0	1	2	≥3	0%	>0% and < 50%	≥50%
All adults	73.0 [70.4–75.4]	27.0 [24.6–29.6]	73.0 [70.4–75.4]	14.9 [13.0–16.9]	7.5 [6.1–9.2]	4.7 [3.6–6.0]	73.0 [70.4–75.4]	14.5 [12.7–16.5]	12.5 [10.8–14.5]
Age (years)									
16–24	66.9 [58.6–74.1]	33.1 [25.9–41.4]	66.9 [58.6–74.1]	14.3 [9.6–20.8]	8.8 [5.1–14.7]	10.0 [5.8–16.8]	66.9 [58.6–74.1]	11.5 [7.1–18.2]	21.6 [15.6–29.2]
25–34	59.8 [52.4–66.8]	40.2 [33.2–47.6]	59.8 [52.4–66.8]	19.9 [14.7–26.3]	12.6 [8.2–18.9]	7.7 [4.5–12.9]	59.8 [52.4–66.8]	25.2 [19.3–32.3]	14.9 [10.4–21]
35–44	67.5 [60.8–73.5]	32.5 [26.5–39.2]	67.5 [60.8–73.5]	18.7 [13.8–24.8]	8.4 [5.1–13.7]	5.4 [3.2–8.8]	67.5 [60.8–73.5]	17.3 [12.7–23.1]	15.2 [10.7–21.0]
45–54	73.0 [66.7–78.5]	27.0 [21.5–33.3]	73.0 [66.7–78.5]	15.7 [11.5–20.9]	7.6 [4.6–12.1]	3.8 [1.9–7.2]	73.0 [66.7–78.5]	18.0 [13.6–23.6]	9.0 [5.8–13.7]
55–64	79.9 [74.3–84.6]	20.1 [15.4–25.7]	79.9 [74.3–84.6]	13.5 [9.6–18.5]	4.1 [2.2–7.6]	2.5 [1.2–5.0]	79.9 [74.3–84.6]	9.6 [6.7–13.5]	10.5 [7.0–15.6]
≥65	85.2 [81.1–88.6]	14.8 [11.4–18.9]	85.2 [81.1–88.6]	9.2 [6.5–12.7]	4.5 [2.8–7.3]	1.0 [0.4–2.9]	85.2 [81.1–88.6]	7.1 [4.9–10.2]	7.6 [5.2–11.1]
Gender									
Men	75.8 [72.3–79.1]	24.2 [20.9–27.7]	75.8 [72.3–79.1]	13.9 [11.4–16.8]	6.6 [4.8–9.0]	3.7 [2.5–5.4]	75.8 [72.3–79.1]	11.1 [8.8–13.9]	13.1 [10.6–16.0]
Women	70.3 [66.6–73.8]	29.7 [26.2–33.4]	70.3 [66.6–73.8]	15.8 [13.2–18.8]	8.4 [6.3–11.0]	5.5 [3.9–7.7]	70.3 [66.6–73.8]	17.8 [15.0–21.0]	11.8 [9.4–14.8]
Occupational social grade									
ABC1 (more advantaged)	74.1 [71.4–76.7]	25.9 [23.3–28.6]	74.1 [71.4–76.7]	15.0 [12.9–17.3]	6.2 [4.9–7.9]	4.7 [3.5–6.2]	74.1 [71.4–76.7]	16.2 [14.1–18.6]	9.7 [8.0–11.6]
C2DE (less advantaged)	71.5 [66.8–75.7]	28.5 [24.3–33.2]	71.5 [66.8–75.7]	14.8 [11.7–18.5]	9.1 [6.5–12.5]	4.7 [2.9–7.4]	71.5 [66.8–75.7]	12.3 [9.3–16.0]	16.2 [13.0–20.2]

Data are presented as percentages with 95% confidence intervals. ABC1 includes managerial, professional, and upper supervisory occupations/C2DE includes manual routine, semi-routine, lower supervisory, long-term unemployed, and state pension.

**Table 2.** Descriptive Data on Vaping Among Participants’ Close Social Connections

	At least one close social connection who vapes		Number of close social connections who vape				Proportion of close social connections who vape		
	No	Yes	0	1	2	≥3	0%	>0% and <50%	≥50%
All adults	74.4 [72.0–76.8]	25.6 [23.2–28.0]	74.4 [72.0–76.8]	15.5 [13.7–17.6]	5.8 [4.7–7.2]	4.2 [3.1–5.6]	74.4 [72.0–76.8]	16.7 [14.7–18.8]	8.9 [7.4–10.6]
Age (years)									
16–24	56.7 [48.5–64.6]	43.3 [35.4–51.5]	56.7 [48.5–64.6]	24.6 [18.4–32.2]	9.7 [5.9–15.4]	9.0 [5.1–15.3]	56.7 [48.5–64.6]	20.0 [14.3–27.2]	23.3 [17.1–30.9]
25–34	61.7 [54.4–68.5]	38.3 [31.5–45.6]	61.7 [54.4–68.5]	18.8 [13.8–25.1]	10.0 [6.6–14.9]	9.5 [5.7–15.5]	61.7 [54.4–68.5]	28.7 [22.5–35.8]	9.6 [6.1–15.0]
35–44	73.8 [67.5–79.3]	26.2 [20.7–32.5]	73.8 [67.5–79.3]	16.3 [11.9–22.1]	7.4 [4.5–11.9]	2.4 [1.1–5.2]	73.8 [67.5–79.3]	16.9 [12.5–22.4]	9.3 [5.9–14.4]
45–54	72.3 [65.6–78.1]	27.7 [21.9–34.4]	72.3 [65.6–78.1]	18.9 [13.9–25.1]	5.3 [3.1–8.9]	3.6 [1.7–7.1]	72.3 [65.6–78.1]	21.4 [16.2–27.7]	6.3 [3.7–10.5]
55–64	83.9 [78.9–87.8]	16.1 [12.2–21.1]	83.9 [78.9–87.8]	10.8 [7.6–15.3]	3.8 [2.1–6.7]	1.5 [0.6–3.7]	83.9 [78.9–87.8]	9.5 [6.6–13.4]	6.6 [4.1–10.6]
≥65	89.5 [85.9–92.3]	10.5 [7.7–14.1]	89.5 [85.9–92.3]	8.4 [5.9–11.7]	1.2 [0.5–2.9]	1.0 [0.3–3.0]	89.5 [85.9–92.3]	7.2 [5.0–10.3]	3.3 [1.8–5.8]
Gender									
Men	77.4 [74.0–80.4]	22.6 [19.6–26.0]	77.4 [74.0–80.4]	13.2 [10.8–16.0]	5.2 [3.8–7.2]	4.2 [2.8–6.2]	77.4 [74.0–80.4]	13.7 [11.3–16.6]	8.9 [6.9–11.4]
Women	71.9 [68.2–75.3]	28.1 [24.7–31.8]	71.9 [68.2–75.3]	17.6 [14.8–20.8]	6.4 [4.8–8.6]	4.1 [2.7–6.3]	71.9 [68.2–75.3]	19.6 [16.7–23.0]	8.5 [6.4–11.1]
Occupational social grade									
ABC1 (more advantaged)	74.5 [71.8–77.1]	25.5 [22.9–28.2]	74.5 [71.8–77.1]	15.6 [13.5–17.9]	5.9 [4.6–7.6]	4.0 [2.9–5.4]	74.5 [71.8–77.1]	17.2 [15.0–19.7]	8.2 [6.7–10.1]
C2DE (less advantaged)	74.3 [69.8–78.4]	25.7 [21.6–30.2]	74.3 [69.8–78.4]	15.5 [12.3–19.4]	5.7 [4.0–8.3]	4.4 [2.6–7.3]	74.3 [69.8–78.4]	15.9 [12.6–19.9]	9.7 [7.2–13.1]

Data are presented as percentages with 95% confidence intervals. ABC1 includes managerial, professional, and upper supervisory occupations/C2DE includes manual routine, semi-routine, lower supervisory, long-term unemployed, and state pension.

Overall, 9.1% of participants reported having no close social connections, 17.1% had one connection, 17.5% had two, 17.5% had three, 11.5% had four, and 27.3% had five or more (**Table S2**). When asked about the smoking and vaping behavior of these connections, 27.0% of participants reported that at least one (14.9% one, 7.5% two, and 4.7% three or more; 12.5% ≥ 50%) connection smokes (**Table 1**) and 25.6% that at least one (15.5% one, 5.8% two, and 4.2% three or more; 8.9% ≥ 50%) vapes (**Table 2**).

Participants aged ≥65 tended to have fewer close social connections than younger participants: among those aged 16–64, the most common number of connections reported was five or more (25.1%–34.6% of participants across age groups), among those aged ≥65 it was one (26.8% of participants; **Table S2**). However, those aged ≥65 were no more likely to have no connections The proportion who reported that at least one connection smokes was highest among those aged 25–34 (40.2%) and lowest among those aged ≥65 (14.8%; **Table 1**). The proportion who reported that at least one connection vapes was highest among those aged 16–24 (43.3%) and lowest among those aged ≥65 (10.5%; **Table 2**). The proportions who reported that at least 50% of their connections smoke or vape were highest among those aged 16–24 (21.6% and 23.3%, respectively) and lowest among those aged ≥65 (7.6% and 3.3%, respectively; **Tables 1 and 2**).

Women were more likely than men to report having four or more close social connections (45.4% vs. 32.5%) and less likely to report having none (6.7% vs. 10.9%) or one (11.3% vs. 23.0%; **Table S2**). The proportion who reported that at least one connection smokes or vapes appeared to be slightly higher among women than men (29.7% vs. 24.2% for smoking, **Table 1**; 28.1% vs. 22.6% for vaping, **Table 2**), although 95% CIs included the possibility of no difference. The proportions who reported that at least one but less than half of their connections smoke or vape were higher among women than men (17.8% vs. 11.1% for smoking, **Table 1**; 19.6% vs. 13.7% for vaping, **Table 2**).

Participants from more advantaged social grades were more likely than those from less advantaged social grades to report having five or more close social connections (32.5% vs. 20.4%) and less likely to report having none (6.4% vs. 12.5%) or one (14.6% vs. 20.4%; **Table S2**). The proportion with at least one connection who smokes was similar among those from less and more advantaged social grades (28.5% vs. 25.9%), but those from less advantaged social grades appeared more likely to report that at least half of their connections smoke (16.2% vs. 9.7%; **Table 1**). There were no notable differences by social grade in the proportion with connections who vape (**Table 2**).

### Associations With Smoking and Vaping Outcomes

**Table 3** summarizes associations of having at least one close social connection who smokes or vapes with smoking and vaping outcomes. **Table S3** summarizes dose–response associations.

**Table 3.** Associations Between Having At Least One Close Social Connection Who Smokes/Vapes and Current Smoking/Vaping, Harm Perceptions of E-cigarettes Versus Cigarettes, and Smoking Cessation Activity

	At least one close social connection who smokes		At least one close social connection who vapes	
	No	Yes	No	Yes
**Current smoking** ^1^				
% [95%CI]	9.4 [7.5 to 11.3]	32.8 [27.6 to 37.9]	—	—
Model 1^a^, OR [95%CI]	Ref	9.07 [6.18 to 13.3]	—	—
Model 2^b^, OR [95%CI]	Ref	7.95 [5.36 to 11.8]	—	—
Model 3^c^, OR [95%CI]	Ref	7.23 [4.74 to 11.0]	—	—
**Current vaping** ^1^				
% [95%CI]	—	—	6.3 [4.7 to 7.9]	29.6 [24.6 to 34.5]
Model 1^a^, OR [95%CI]	—	—	Ref	9.08 [6.02 to 13.7]
Model 2^d^, OR [95%CI]	—	—	Ref	4.97 [3.14 to 7.86]
Model 3^c^, OR [95%CI]	—	—	Ref	5.16 [3.15 to 8.43]
**Perception of e-cigarettes as less harmful than cigarettes** **^2^**				
% [95%CI]	15.1 [7.2 to 23.0]	22.5 [14.7 to 30.2]	12.2 [6.8 to 17.6]	30.8 [19.9 to 41.7]
Model 1^a^, OR [95%CI]	Ref	1.29 [0.46 to 3.58]	Ref	3.65 [1.69 to 7.89]
Model 2^e^, OR [95%CI]	Ref	0.77 [0.22 to 2.66]	Ref	2.19 [0.83 to 5.75]
Model 3^c^, OR [95%CI]	Ref	0.63 [0.16 to 2.45]	Ref	2.37 [0.82 to 6.90]
**Attempts to quit smoking** **^3^**				
% [95%CI]	34.3 [24.9 to 43.7]	35.7 [26.8 to 44.5]	34.3 [25.9 to 42.7]	36.4 [26.2 to 46.7]
Model 1^a^, OR [95%CI]	Ref	1.22 [0.59 to 2.54]	Ref	1.29 [0.67 to 2.50]
Model 2^b^, OR [95%CI]	Ref	1.03 [0.48 to 2.20]	Ref	0.93 [0.45 to 1.93]
Model 3^c^, OR [95%CI]	Ref	1.05 [0.47 to 2.35]	Ref	0.92 [0.43 to 1.99]
**Smoking cessation** **^3^**				
% [95%CI]	19.8 [12.3 to 27.2]	12.2 [6.6 to 17.8]	14.3 [8.8 to 19.7]	17.9 [9.9 to 25.8]
Model 1^a^, OR [95%CI]	Ref	0.44 [0.18 to 1.11]	Ref	1.39 [0.61 to 3.15]
Model 2^f^, OR [95%CI]	Ref	0.54 [0.22 to 1.33]	Ref	1.63 [0.68 to 3.91]
Model 3^c^, OR [95%CI]	Ref	0.46 [0.17 to 1.23]	Ref	1.98 [0.72 to 5.39]
**Use of e-cigarettes as a smoking cessation aid** **^4^**				
% [95%CI]	40.0 [23.2 to 56.9]	40.1 [24.8 to 55.3]	27.9 [14.5 to 41.3]	57.0 [39.3 to 74.6]
Model 1^a^, OR [95%CI]	Ref	1.07 [0.29 to 3.88]	Ref	8.49 [1.97 to 36.5]
Model 2^f^, OR [95%CI]	Ref	0.94 [0.19 to 4.72]	Ref	17.9 [1.80 to 178]
Model 3^c^, OR [95%CI]	Ref	0.89 [0.21 to 3.77]	Ref	18.0 [1.80 to 181]

^1^Among adults (unweighted *n* = 1618).^2^ Among current smokers (*n* = 234).^3^ Among past-year smokers (*n* = 278).^4^ Among past-year smokers who tried to quit (*n* = 89).

^a^Adjusted for the total number of close social connections.

^b^Model 1 plus an additional adjustment for age, gender, and occupational social grade.

^c^Model 2 plus an additional adjustment for having at least one close social connection who engages in the other behavior (ie, vaping, for analyses of associations with having at least one close social connection who smokes, and vice versa).

^d^Model 1 plus an additional adjustment for age, gender, occupational social grade, and smoking status.

^e^Model 1 plus an additional adjustment for age, gender, occupational social grade, and vaping status.

^f^Model 1 plus an additional adjustment for age, gender, occupational social grade, and level of cigarette addiction.

Each association was tested in a separate model.

Participants with at least one close social connection who smokes were more likely than those with none to smoke themselves (32.8% vs. 9.4%; **Table 3**). The prevalence of current smoking was greater among those with a greater proportion of connections who smoke (46.2% for those with 50% or more vs. 21.2% for those with at least one and less than 50%; **Table S3**). After adjustment for the total number of close social connections, sociodemographic characteristics, and vaping among connections, those with at least one connection who smokes had 7.23 times higher odds of being a smoker than those with none (**Table 3**), while those with one, two, and three or more connections who smoke had 5.16, 12.0, and 20.3 times higher odds, respectively (**Table S3**). There were no clear age differences in the strength of the association between having at least one connection who smokes and currently smoking (**Table S4**); including the interaction term did not improve the model fit (AIC = 1179 with the interaction term and 1170 without).

Participants with at least one close social connection who vapes were more likely than those with none to vape themselves (29.6% vs. 6.3%; **Table 3**). The prevalence of current vaping was greater among those with a greater proportion of connections who vape (38.4% for those with 50% or more vs. 24.9% for those with at least one and less than 50%; **Table S3**). After adjustment for the total number of connections, sociodemographic characteristics, smoking status, and smoking among connections, those with at least one connection who vapes had 5.16 times higher odds of being a vaper than those with none (**Table 3**). There were no clear age differences in the strength of the association between having at least one connection who vapes and current vaping (**Table S4**); including the interaction term did not improve the model fit (AIC = 833 with the interaction term and 825 without).

Among current smokers, there was no clear association between smoking among close social connections and harm perceptions of e-cigarettes relative to cigarettes (**Table 3**). However, those with at least one connection who vapes were more likely to perceive e-cigarettes as less harmful than cigarettes (30.8% vs. 12.2%; **Table 3**). This proportion was not greater for those with a greater proportion of connections who vape (**Table S3**). After adjustment for the total number of connections, sociodemographic characteristics, vaping status, and smoking among connections, those with at least one connection who vapes had 2.37 times higher odds of perceiving e-cigarettes as less harmful than cigarettes (**Table 3**), but the 95% CI included the possibility of no difference.

Among past-year smokers, the proportion who attempted to quit smoking in the past year was similar among those with and without close social connections who smoke (35.7% vs. 34.3%) or vape (36.4% vs. 34.3%; **Table 3**).

Among past-year smokers, the proportion who reported stopping smoking in the past year was slightly lower among those with at least one close social connection who smokes than those without (12.2% vs. 19.8%; **Table 3**) but was more similar among those with and without connections who vape (17.9% vs. 14.3%; **Table 3**). After adjustment for the total number of connections, sociodemographic characteristics, and vaping among connections, those with at least one connection who smokes had lower odds of smoking cessation (OR = 0.46), but the 95%CI was wide and included the possibility of no difference (**Table 3**). Point estimates trended in the opposite direction for associations between vaping among connections and cessation (eg, OR = 1.98 for those with at least one vs. no close social connections who vape), but the 95%CI included the possibility of no difference (**Table 3**).

Among past-year smokers who tried to quit, the proportion who reported using e-cigarettes as a smoking cessation aid was similar among those with and without close social connections who smoke (40.1% vs. 40.0%; **Table 3**). However, those with at least one connection who vapes were more likely than those with none to use e-cigarettes in their most recent attempt to quit (57.0% vs. 27.9%; **Table 3**). The proportion who reported using e-cigarettes as a smoking cessation aid was greater for those with a greater proportion of connections who vape (81.3% for those with 50% or more vs. 40.9% for those with at least one and less than 50%; **Table S3**). After adjusting for the total number of connections, sociodemographic characteristics, level of cigarette addiction, and smoking among connections, those with at least one connection who vapes had 18.0 times higher odds of using e-cigarettes to quit smoking than those with none (**Table 3**). We note that the 95% CI was extremely wide. We did not analyze dose–response associations due to the small sample size for analysis of this outcome (*n* = 89).

## Discussion

In November 2023, similar proportions of adults (≥16 years) in England reported having at least one close social connection who smokes (27.0%) or vapes (25.6%). Those with connections who smoke were more likely than those without to smoke themselves, and potentially less likely to quit. Those with connections who vape were more likely than those without to vape themselves, to perceive e-cigarettes as less harmful than cigarettes, and to use e-cigarettes in their most recent attempt to quit smoking.

There were differences across sociodemographic groups in the extent of smoking and vaping among participants’ close social connections. Older adults (≥65 years) were less likely than younger age groups to have connections who smoke or vape, and men appeared slightly less likely to than women. This was probably due, at least in part, to older adults and men tending to report having fewer close social connections overall, although the pattern of results held when analyzing the proportion of connections. There was a different pattern by socioeconomic position: those from less advantaged social grades tended to have fewer close social connections than those who were more advantaged but were similarly likely to have at least one connection who smokes or vapes and more likely to report that at least 50% of their connections smoke. This is consistent with rates of vaping and particularly smoking being higher among less advantaged socioeconomic groups.^7,34^

We observed strong associations between smoking and vaping among close social connections and the odds of participants engaging in the same behavior. Adults with at least one close social connection who smokes had 7 times higher odds of being a smoker themselves and there was evidence of a dose–response association (whereby the odds of smoking increased with the number/proportion of connections who smoke). Associations with vaping appeared slightly weaker than for smoking (5 times higher odds among those with at least one close social connection who vapes) and with a less clear dose–response association. Further research (eg, qualitative) is needed to understand more about these potential differences. It is possible that vaping does not (yet) have such a strong social and cultural identity as smoking,^21–23^ meaning it does not spread through social networks to the same extent. Alternatively, it may be that vaping is a less dependence-forming behavior,^35,36^ making it easier for people who try vaping as a result of social influence to quit than those who try smoking. Associations between smoking and vaping among close social connections and the odds of engaging in the same behavior did not show a clear pattern across age groups, indicating social network influences on smoking and vaping may be similar across adulthood. However, we note that the sample size was relatively small and there may be differences we were not able to detect, so more data are needed to confirm these findings. Further research could also explore differences by other sociodemographic variables, such as gender and socioeconomic position.

Previous research has shown that the chance that a person smokes is reduced if their close social connections quit smoking and that people tend to quit in concert.^1^ Consistent with this, our results provide some evidence that smokers may be less likely to quit if they have close social connections who smoke, although sample sizes were small and the results uncertain. We did not see the same association with vaping among close social connections, suggesting vaping does not undermine other people stopping smoking. This is in line with previous findings that smokers who are regularly exposed to other people using e-cigarettes are neither less likely to be motivated to quit nor less likely to attempt to do so.^37^ In fact, vaping among close social connections could potentially have benefits for smokers. The data suggest those with connections who vape were more likely to hold accurate perceptions of the relative harms of e-cigarettes versus cigarettes and to report using e-cigarettes to support quit attempts. E-cigarettes are one of the most effective interventions for smoking cessation^38^ so an increase in the proportion of smokers using them may lead to more successful quit attempts.^39^ Although we did not observe a clear association between vaping among close social connections and smoking cessation, point estimates favored a potential benefit. The data suggested this may not be a linear relationship (ie, the more connections who are vapers, the better) but may be more nuanced (eg, a person who smokes may only need to have one or two connections who vape to have higher odds of quitting). It would be interesting to look at this with a larger sample size of past-year smokers to explore whether the same pattern is observed.

There are two potential explanations for the observed associations between people’s vaping and smoking status and those of their close connections. Either people seek out others with behaviors similar to their own (social selection), or other people’s behavior influences their own (social influence).^40^ If the latter is the case, then interventions that aim to reduce the uptake of smoking or vaping or encourage smokers to switch to vaping may have spillover effects experienced by individuals not intentionally targeted by the intervention. Based on our findings, it seems likely that any such effects will be largely positive. Therefore, evaluations of initiatives such as the UK government’s Swap to Stop campaign (which aims to provide a million smokers with a free e-cigarette starter kit and behavioral support) should consider indirect effects on the social networks of those who receive the intervention as well as direct effects on recipients.

This study had several limitations. The cross-sectional design means we cannot determine the temporality of potential effects. Further work to disentangle the direction of associations (ie, social selection vs. social influence) would require detailed individual-level longitudinal data on smoking and vaping behaviors, sociodemographic attributes, and social connections. Questions on social connections were only included in a single wave of data collection, so the sample size was relatively small—particularly for analyses within subgroups (eg, among past-year smokers who made a quit attempt). We therefore cannot rule out the possibility that there are other associations that we were unable to detect with our data. The analysis of the association between having close social connections who vape and the use of e-cigarettes as a smoking cessation aid yielded a large effect size with an extremely wide 95% CI, so should be interpreted with some caution.^41^ In our analyses, we did not distinguish between different types of close social connection (eg, partner vs. friend) and it is possible associations may differ according to the nature of the relationship (eg, living together, frequency of contact). All data were self-reported and quitting outcomes relied on recall of the past year. However, we would not expect recall bias to differ according to smoking or vaping among close social connections, so this should not substantially affect our results. Our analyses do not consider the dual use of smoking and vaping, which is common,^42^ among either close social connections or participants; this would be an interesting direction for future research. Finally, findings may not generalize beyond England to other countries with different social structures, smoking and vaping norms, or regulatory environments.

In conclusion, we replicated well-established associations with smoking and found similar evidence for vaping. Having close social connections who smoke or vape is strongly associated with engaging in the same behavior. However, dose–response associations may be stronger for smoking than vaping. People may also be more likely to perceive e-cigarettes as less harmful than cigarettes if they have close social connections who vape, and are more likely to use e-cigarettes to quit smoking. Interventions that encourage smokers to switch to vaping may have positive spillover effects on social connections’ perceptions of e-cigarettes and the use of these products to support smoking cessation.

## Supplementary material

Supplementary material is available at *Nicotine and Tobacco Research* online.

ntae225_suppl_Supplementary_Tables_S1-S4

## Data Availability

The data used in these analyses are available on Open Science Framework (https://osf.io/hvxcp/).
