# Supplementary material for: Associations of Close Social Connections With Smoking and Vaping: A Population Study in England
Source: Nicotine Tob Res. 2024 Sep 24;27(3):447–56. doi: 10.1093/ntr/ntae225 (PMC11847782; doi:10.1093/ntr/ntae225)
Supplement: ntae225_suppl_Supplementary_Tables_S1-S4 [file ntae225_suppl_supplementary_tables_s1-s4.docx]

## Table S1. Weighted sample characteristics

|  | **%^1^** |
| --- | --- |
|  |  |
| Age (years)^2^ |  |
| Mean (SD) | 48.0 (18.3) |
| 16-24 | 12.8 [11.0–14.8] |
| 25-34 | 17.0 [14.9–19.3] |
| 35-44 | 16.1 [14.3–18.1] |
| 45-54 | 15.7 [13.9–17.8] |
| 55-64 | 15.6 [13.8–17.5] |
| ≥65 | 22.8 [20.7–25.0] |
|  |  |
| Gender^2^ |  |
| Men | 48.5 [45.8–51.2] |
| Women | 50.8 [48.1–53.5] |
| Non-binary | 0.7 [0.4–1.3] |
| *Missing* | 9 |
|  |  |
| Occupational social grade C2DE (less advantaged)^2^ | 43.7 [40.9–46.5] |
|  |  |
| Smoking status^2^ |  |
| Never smoker | 57.8 [55.2–60.5] |
| Former smoker | 26.3 [24.0–28.7] |
| Current smoker | 15.9 [13.9–18.0] |
| *Missing* | 2 |
|  |  |
| Current vaper^2^ | 12.1 [10.4–14.0] |
|  |  |
| Perceive e-cigarettes as less harmful than cigarettes^3^ | 18.9 [14.0–25.0] |
|  |  |
| Tried to quit in past year^4^ | 35.0 [28.9–41.6] |
| *Missing* | 17 |
|  |  |
| Stopped smoking in past year^4^ | 15.4 [11.4–20.5] |
|  |  |
| Used e-cigarette in most recent quit attempt^5^ | 39.6 [29.1–51.2] |
|  |  |

^1^ Data are shown as valid weighted column percentages with 95% confidence intervals, unless otherwise specified. Sample sizes (including numbers of missing cases) are unweighted.

^2^ Among adults (*n*=1,618).

^3^ Among current smokers (*n*=234).

^4^ Among past-year smokers (*n*=278).

^5^ Among past-year smokers who tried to quit (*n*=89).

## Table S2. Total number of close social connections within sociodemographic subgroups

|  | **Total number of close social connections** | | | | | | |
| --- | --- | --- | --- | --- | --- | --- | --- |
|  | **0** | **1** | **2** | **3** | **4** | **≥5** |  |
|  |  |  |  |  |  |  |  |
| All adults | 9.1 [7.4–10.7] | 17.1 [15.1–19.1] | 17.5 [15.5–19.6] | 17.5 [15.4–19.5] | 11.5 [9.9–13.2] | 27.3 [24.9–29.6] |  |
|  |  |  |  |  |  |  |  |
| Age (years) |  |  |  |  |  |  |  |
| 16-24 | 10.9 [5.5–16.3] | 12.6 [6.9–18.2] | 22.4 [15.7–29.2] | 15.6 [10.1–21.1] | 13.4 [7.3–19.6] | 25.1 [18.2–31.9] |  |
| 25-34 | 9.2 [4.9–13.4] | 10.3 [6.1–14.4] | 16.7 [11.1–22.3] | 15.5 [10.5–20.4] | 13.8 [8.9–18.7] | 34.6 [27.7–41.5] |  |
| 35-44 | 7.6 [4.2–10.9] | 10.8 [6.9–14.7] | 21.1 [15.7–26.5] | 22.7 [16.8–28.6] | 12.7 [8.7–16.8] | 25.1 [19.6–30.6] |  |
| 45-54 | 10.0 [5.3–14.6] | 18.0 [12.6–23.4] | 12.4 [8.0–16.7] | 20.0 [14.3–25.6] | 10.0 [6.4–13.7] | 29.7 [23.5–35.9] |  |
| 55-64 | 11.4 [6.7–16.1] | 19.7 [14.8–24.7] | 14.7 [9.8–19.5] | 13.9 [9.4–18.3] | 9.9 [6.4–13.4] | 30.4 [24.7–36.0] |  |
| ≥65 | 6.8 [4.3–9.3] | 26.8 [22.1–31.6] | 18.4 [14.3–22.6] | 17.1 [13.2–20.9] | 10.2 [7.2–13.2] | 20.7 [16.5–24.9] |  |
|  |  |  |  |  |  |  |  |
| Gender |  |  |  |  |  |  |  |
| Men | 10.9 [8.4–13.3] | 23.0 [19.8–26.2] | 16.0 [13.2–18.7] | 17.6 [14.7–20.6] | 8.6 [6.6–10.6] | 23.9 [20.6–27.2] |  |
| Women | 6.7 [4.6–8.9] | 11.3 [8.9–13.7] | 19.2 [16.1–22.4] | 17.3 [14.4–20.2] | 14.4 [11.7–17.1] | 31.0 [27.6–34.5] |  |
|  |  |  |  |  |  |  |  |
| Occupational social grade |  |  |  |  |  |  |  |
| ABC1 (more advantaged) | 6.4 [5.0–7.8] | 14.6 [12.5–16.7] | 15.9 [13.7–18.0] | 17.7 [15.5–20.0] | 12.9 [10.9–14.9] | 32.5 [29.7–35.4] |  |
| C2DE (less advantaged) | 12.5 [9.3–15.8] | 20.4 [16.6–24.1] | 19.7 [15.8–23.6] | 17.1 [13.5–20.8] | 9.8 [7.0–12.6] | 20.4 [16.5–24.4] |  |
|  |  |  |  |  |  |  |  |

ABC1 includes managerial, professional and upper supervisory occupations / C2DE includes manual routine, semi-routine, lower supervisory, long-term unemployed, and state pension.

## Table S3. Associations between the number and proportion of close social connections who smoke/vape and current smoking/vaping, harm perceptions of e-cigarettes vs cigarettes, and smoking cessation activity

|  | **Number of close social connections who smoke** | | | |  | **Proportion of close social connections who smoke** | | |
| --- | --- | --- | --- | --- | --- | --- | --- | --- |
|  | **0** | **1** | **2** | **≥3** |  | **0%** | **>0% and <50%** | **≥50%** |
|  |  |  |  |  |  |  |  |  |
| **Current smoking^1^** |  |  |  |  |  |  |  |  |
| % [95%CI] | 9.4 [7.5–11.3] | 26.2 [19.8–32.6] | 40.2 [29.7–50.8] | 41.8 [28.9–54.7] |  | 9.4 [7.5–11.3] | 21.2 [15.3–27.1] | 46.2 [38.1–54.2] |
| Model 1^a^, OR [95%CI] | Ref | 6.08 [3.88–9.51] | 15.2 [8.73–26.6] | 25.3 [12.0–52.8] |  | Ref | 5.14 [3.08–8.57] | 12.7 [8.18–19.6] |
| Model 2^b^, OR [95%CI] | Ref | 5.51 [3.48–8.73] | 12.9 [7.33–22.7] | 19.8 [9.24–42.2] |  | Ref | 4.68 [2.74–7.98] | 10.7 [6.87–16.8] |
| Model 3^c^, OR [95%CI] | Ref | 5.16 [3.20–8.32] | 12.0 [6.60–21.8] | 20.3 [9.00–45.9] |  | Ref | 4.36 [2.50–7.58] | 9.81 [6.10–15.8] |
|  |  |  |  |  |  |  |  |  |
| **Perception of e-cigarettes as less harmful than cigarettes^2^** |  |  |  |  |  |  |  |  |
| % [95%CI] | 15.1 [7.2–23.0] | 29.6 [16.4–42.9] | 16.6 [5.8–27.4] | 17.3 [2.8–31.8] |  | 15.1 [7.2–23.0] | 30.1 [15.3–44.9] | 18.4 [9.9–27.0] |
| Model 1^a^, OR [95%CI] | Ref | 2.03 [0.76–5.41] | 0.73 [0.23–2.37] | 0.70 [0.13–3.67] |  | Ref | 2.06 [0.43–9.74] | 1.06 [0.38–2.99] |
| Model 2^d^, OR [95%CI] | Ref | 1.55 [0.46–5.22] | 0.36 [0.08–1.60] | 0.27 [0.04–1.87] |  | Ref | 1.48 [0.24–9.18] | 0.55 [0.15–2.05] |
| Model 3^c^, OR [95%CI] | Ref | 1.48 [0.32–6.86] | 0.21 [0.04–1.18] | 0.20 [0.02–1.74] |  | Ref | 1.24 [0.18–8.34] | 0.44 [0.11–1.85] |
|  |  |  |  |  |  |  |  |  |
| **Attempts to quit smoking^3^** |  |  |  |  |  |  |  |  |
| % [95%CI] | 34.3 [24.9–43.7] | 38.2 [24.8–51.6] | 32.3 [16.2–48.4] | 35.4 [18.4–52.5] |  | 34.3 [24.9–43.7] | 29.6 [15.7–43.5] | 39.0 [27.7–50.3] |
| Model 1^a^, OR [95%CI] | Ref | 1.23 [0.55–2.76] | 1.06 [0.38–2.91] | 1.54 [0.49–4.89] |  | Ref | 1.02 [0.36–2.89] | 1.29 [0.61–2.74] |
| Model 2^b^, OR [95%CI] | Ref | 1.16 [0.49–2.75] | 0.91 [0.34–2.46] | 0.78 [0.23–2.64] |  | Ref | 1.10 [0.35–3.46] | 1.01 [0.46–2.22] |
| Model 3^c^, OR [95%CI] | Ref | 1.17 [0.47–2.88] | 0.96 [0.34–2.70] | 0.84 [0.24–2.88] |  | Ref | 1.17 [0.35–3.89] | 1.09 [0.48–2.48] |
|  |  |  |  |  |  |  |  |  |
| **Smoking cessation^3^** |  |  |  |  |  |  |  |  |
| % [95%CI] | 19.8 [12.3–27.2] | 16.1 [6.5–25.7] | 4.6 [0.0–9.9] | 14.8 [2.6–27.1] |  | 19.8 [12.3–27.2] | 14.8 [5.4–24.2] | 10.8 [3.9–17.7] |
| Model 1^a^, OR [95%CI] | Ref | 0.60 [0.23–1.58] | 0.14 [0.03–0.61] | 0.55 [0.13–2.25] |  | Ref | 0.52 [0.16–1.73] | 0.41 [0.15–1.14] |
| Model 2^e^, OR [95%CI] | Ref | 0.66 [0.25–1.73] | 0.22 [0.06–0.83] | 0.78 [0.15–4.04] |  | Ref | 0.55 [0.15–2.03] | 0.53 [0.21–1.37] |
| Model 3^c^, OR [95%CI] | Ref | 0.55 [0.20–1.57] | 0.14 [0.03–0.57] | 0.84 [0.16–4.40] |  | Ref | 0.48 [0.12–1.85] | 0.47 [0.17–1.33] |
|  |  |  |  |  |  |  |  |  |
|  | **Number of close social connections who vape** | | | |  | **Proportion of close social connections who vape** | | |
|  | **0** | **1** | **2** | **≥3** |  | **0%** | **>0% and <50%** | **≥50%** |
|  |  |  |  |  |  |  |  |  |
| **Current vaping^1^** |  |  |  |  |  |  |  |  |
| % [95%CI] | 6.3 [4.7–7.9] | 26.2 [20.0–32.4] | 37.6 [27.0–48.3] | 30.9 [18.1–43.7] |  | 6.3 [4.7–7.9] | 24.9 [19.1–30.7] | 38.4 [29.3–47.4] |
| Model 1^a^, OR [95%CI] | Ref | 7.43 [4.68–11.8] | 14.6 [8.13–26.2] | 12.9 [6.22–26.6] |  | Ref | 7.21 [4.41–11.8] | 11.2 [6.82–18.5] |
| Model 2^f^, OR [95%CI] | Ref | 4.16 [2.48–6.99] | 7.86 [4.08–15.2] | 6.01 [2.43–14.9] |  | Ref | 4.21 [2.46–7.21] | 5.84 [3.17–10.7] |
| Model 3^c^, OR [95%CI] | Ref | 4.29 [2.49–7.37] | 8.47 [4.24–16.9] | 6.30 [2.37–16.7] |  | Ref | 4.40 [2.53–7.65] | 6.00 [3.15–11.4] |
|  |  |  |  |  |  |  |  |  |

*Table continues on next page.*

**Table S3.** *continued*

|  | **Number of close social connections who vape** | | | |  | **Proportion of close social connections who vape** | | |
| --- | --- | --- | --- | --- | --- | --- | --- | --- |
|  | **0** | **1** | **2** | **≥3** |  | **0%** | **>0% and <50%** | **≥50%** |
|  |  |  |  |  |  |  |  |  |
| **Perception of e-cigarettes as less harmful than cigarettes^2^** |  |  |  |  |  |  |  |  |
| % [95%CI] | 12.2 [6.8–17.6] | 26.5 [11.7–41.3] | 40.9 [18.6–63.3] | 30.4 [7.2–53.7] |  | 12.2 [6.8–17.6] | 31.9 [16.6–47.2] | 29.3 [14.4–44.3] |
| Model 1^a^, OR [95%CI] | Ref | 2.89 [1.14–7.32] | 6.24 [1.97–19.7] | 4.06 [1.01–16.2] |  | Ref | 4.35 [1.49–12.8] | 3.25 [1.32–7.97] |
| Model 2^d^, OR [95%CI] | Ref | 1.59 [0.44–5.71] | 4.41 [1.21–16.0] | 2.38 [0.45–12.6] |  | Ref | 2.31 [0.66–8.09] | 2.09 [0.70–6.20] |
| Model 3^c^, OR [95%CI] | Ref | 1.62 [0.40–6.55] | 7.48 [1.75–32.0] | 5.00 [0.85–29.5] |  | Ref | 2.56 [0.65–10.1] | 2.37 [0.76–7.35] |
|  |  |  |  |  |  |  |  |  |
| **Attempts to quit smoking^3^** |  |  |  |  |  |  |  |  |
| % [95%CI] | 34.3 [25.9–42.7] | 38.4 [24.6–52.2] | 35.4 [15.7–55.2] | 31.3 [8.0–54.6] |  | 34.3 [25.9–42.7] | 38.1 [24.1–52.1] | 34.2 [19.5–48.9] |
| Model 1^a^, OR [95%CI] | Ref | 1.32 [0.63–2.77] | 1.26 [0.46–3.47] | 1.21 [0.34–4.27] |  | Ref | 1.97 [0.80–4.86] | 0.97 [0.42–2.23] |
| Model 2^b^, OR [95%CI] | Ref | 1.00 [0.44–2.24] | 0.92 [0.30–2.81] | 0.60 [0.15–2.36] |  | Ref | 1.65 [0.63–4.31] | 0.60 [0.24–1.50] |
| Model 3^c^, OR [95%CI] | Ref | 0.96 [0.41–2.25] | 0.91 [0.29–2.85] | 0.63 [0.16–2.50] |  | Ref | 1.63 [0.61–4.35] | 0.58 [0.22–1.52] |
|  |  |  |  |  |  |  |  |  |
| **Smoking cessation^3^** |  |  |  |  |  |  |  |  |
| % [95%CI] | 14.3 [8.8–19.7] | 19.3 [8.5–30.2] | 22.7 [5.5–39.9] | 5.2 [0.0–15.3] |  | 14.3 [8.8–19.7] | 18.7 [8.0–29.3] | 16.7 [4.8–28.7] |
| Model 1^a^, OR [95%CI] | Ref | 1.35 [0.53–3.44] | 2.28 [0.71–7.35] | 0.37 [0.04–3.50] |  | Ref | 1.61 [0.55–4.68] | 1.24 [0.44–3.47] |
| Model 2^e^, OR [95%CI] | Ref | 1.46 [0.56–3.84] | 3.05 [0.81–11.4] | 0.43 [0.06–2.93] |  | Ref | 2.23 [0.76–6.58] | 1.20 [0.38–3.76] |
| Model 3^c^, OR [95%CI] | Ref | 1.87 [0.61–5.71] | 5.26 [1.04–26.6] | 0.40 [0.06–2.81] |  | Ref | 2.61 [0.81–8.35] | 1.52 [0.42–5.47] |
|  |  |  |  |  |  |  |  |  |

^1^ Among adults (unweighted *n*=1,618). ^2^ Among current smokers (*n*=234). ^3^ Among past-year smokers (*n*=278). ^4^ Among past-year smokers who tried to quit (*n*=89).

^a^ Adjusted for the total number of close social connections.

^b^ Model 1 plus additional adjustment for age, gender, and occupational social grade.

^c^ Model 2 plus additional adjustment for having at least one close social connection who engages in the other behaviour (i.e., vaping, for analyses of associations with having at least one close social connection who smokes, and vice versa).

^d^ Model 1 plus additional adjustment for age, gender, occupational social grade, and vaping status.

^e^ Model 1 plus additional adjustment for age, gender, occupational social grade, and level of cigarette addiction.

^f^ Model 1 plus additional adjustment for age, gender, occupational social grade, and smoking status.

Each association was tested in a separate model.

## Table S4. Interactions with age

|  | **At least one close social connection who smokes, OR [95%CI]^1^** | **At least one close social connection who vapes, OR [95%CI]^2^** |
| --- | --- | --- |
|  |  |  |
| **Current smoking** |  |  |
| Intercept | 0.45 [0.21–0.97] | - |
| Interaction with age |  | - |
| 16-24 | Ref | - |
| 25-34 | 1.49 [0.92–2.25] | - |
| 35-44 | 1.65 [0.51–5.35] | - |
| 45-54 | 0.89 [0.26–3.03] | - |
| 55-64 | 1.99 [0.57–6.87] | - |
| ≥65 | 1.01 [0.30–3.45] | - |
|  |  |  |
| **Current vaping** |  |  |
| Intercept | - | 0.04 [0.01–0.15] |
| Interaction with age | - |  |
| 16-24 | - | Ref |
| 25-34 | - | 1.32 [0.28–6.25] |
| 35-44 | - | 0.69 [0.15–3.17] |
| 45-54 | - | 1.81 [0.33–9.82] |
| 55-64 | - | 0.45 [0.08–2.49] |
| ≥65 | - | 0.75 [0.13–4.31] |
|  |  |  |

^1^ Adjusted for the total number of close social connections, age, gender, occupational social grade, and having at least one close social connection who vapes.

^2^ Adjusted for the total number of close social connections, age, gender, occupational social grade, smoking status, and having at least one close social connection who smokes.
